# Outstanding Properties of the Hydration Shell around β-d-Glucose: A Computational Study

**DOI:** 10.1021/acsomega.4c00798

**Published:** 2024-04-25

**Authors:** Imre Bakó, László Pusztai, Szilvia Pothoczki

**Affiliations:** † HUN-REN Research Centre for Natural Sciences, Magyar tudósok körútja 2., H-1117 Budapest, Hungary; ‡ HUN-REN Wigner Research Centre for Physics, Konkoly-Thege M. út 29-33., H-1121 Budapest, Hungary; § International Research Organization for Advanced Science and Technology (IROAST), Kumamoto University, 2-39-1 Kurokami, Chuo-ku, Kumamoto 860-8555, Japan

## Abstract

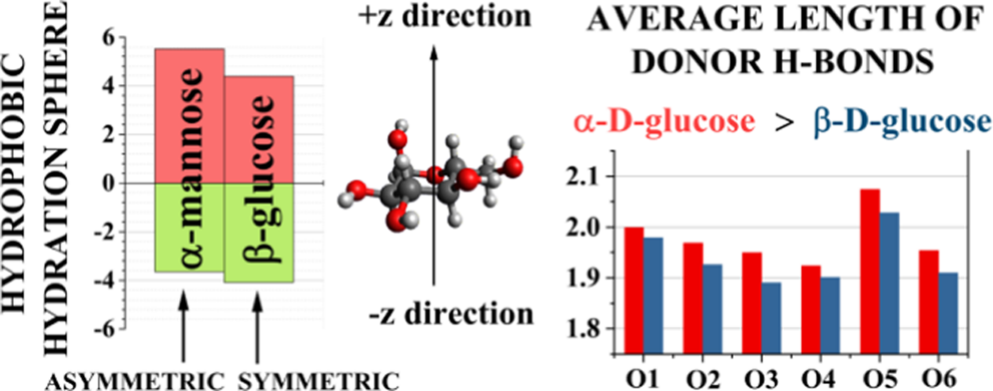

Ab initio molecular dynamics (AIMD) simulations have been performed on aqueous solutions of four simple sugars, α-d-glucose, β-d-glucose, α-d-mannose, and α-d-galactose. Hydrogen-bonding (HB) properties, such as the number of donor- and acceptor-type HB-s, and the lengths and strengths of hydrogen bonds between sugar and water molecules, have been determined. Related electronic properties, such as the dipole moments of water molecules and partial charges of the sugar O atoms, have also been calculated. The hydrophilic and hydrophobic shells were characterized by means of spatial distribution functions. β-d-Glucose was found to form the highest number of hydrophilic and the smallest number of hydrophobic connections to neighboring water molecules. The average sugar–water H-bond length was the shortest for β-d-glucose, which suggests that these are the strongest such H-bonds. Furthermore, β-d-glucose appears to stand out in terms of the symmetry properties of both its hydrophilic and hydrophobic hydration shells. In summary, in all aspects considered here, there seems to be a correlation between the distinct characteristics of β-d-glucose reported here and its outstanding solubility in water. Admittedly, our findings represent only some of the important factors that influence the solubility.

## Introduction

Carbohydrates are one of the most crucial biomolecules that play a principal role in several biological processes such as molecular recognition, structural stabilization, and modification of proteins and nucleic acids. They also act as cryoprotective molecules in living cells.^1^ Additionally, they play a significant role in many industrial applications related to, e.g., the food, biotechnology, biofuel, and cosmetic industries.^1−3^ The most important, and therefore the most studied, of these are hexopyranose sugars. Their five chirality centers lead to 32 diastereoisomers characterized by the axial or equatorial orientation of ring substituents.

Many important properties derive from the conformational flexibility and hydroxymethyl and exocyclic hydroxyl group orientations and their interaction with water. The interconversion between conformers is hindered by high free-energy barriers, leading to characteristic times on the order of hundred picoseconds to microseconds.^4,5^ This long time is one of the problems while constructing appropriate force fields for molecular dynamics (MD) or Monte Carlo (MC) simulations.^4−6^

Although carbohydrates are generally considered hydrophilic compounds, they have substantial hydrophobicity that varies with their structure. In the gas phase, the energetically most stable conformations are the ones whose hydroxyl groups form a well-defined intramolecular H-bonded pattern.^7,8^ The competition between these intramolecular H-bonds and the intermolecular ones that are formed between the oxygens of sugars and water molecules, together with hydrophobic interactions, determines the solvation shell of these molecules.

Various theoretical^9−22^ and experimental techniques^23−33^ have been used to explore and understand the relationship between the conformational and configurational properties of carbohydrates and the structural arrangement of water molecules in the first hydration shell at the atomistic level. Approximately 10–12 water molecules are bonded to a central hexopyranose monosaccharide molecule via H-bonds according to classical and ab initio MD simulations and neutron diffraction results.^20,21,23,24^ Additionally, 8–10 water molecules are attached through significantly weaker interactions to the hydrophobic parts (“surface”) of the sugar molecules.^23,24^ The energetic properties of these water molecules are determined through van der Waals and CH···O_water_-type interactions. Therefore, it is important to consider the polarizability and charge transfer properties of the alcoholic groups of sugar and water molecules when describing interactions between them.

**Figure 1:**
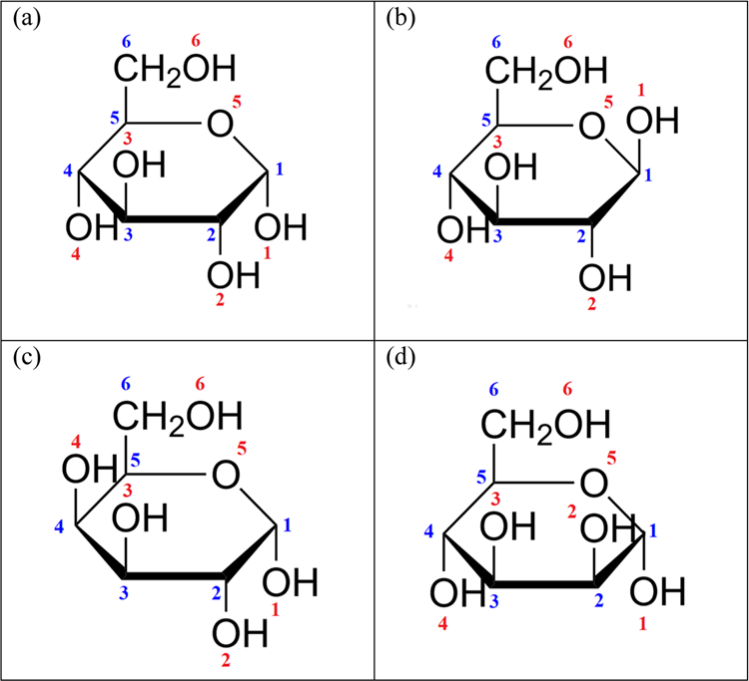
Haworth projections of (a) α-d-glucose, (b) β-d-glucose, (c) α-d-galactose, and (d) α-d-mannose.

Here, we consider four simple sugars, α-d-glucose, β-d-glucose, α-d-galactose, and α-d-mannose, that possess very similar molecular structures (Figure 1) but quite different water solubilities (Table 1). A major focus of the present work is to find whether this property may be related to the hydrogen-bonding environment of sugar molecules. It is, however, important to note here that the H-bond interaction is only one of the important factors associated with solubility. Other types of interactions, e.g., those between sugar molecules in the crystalline phase, changes in terms of the magnitude of water–water interactions, as well as solute-induced changes in the hydration sphere of water molecules, might well also play important roles in the process.

**Table 1:** Water Solubilities of the Hexopyranose Monosaccharides Considered Here (https://pubchem.ncbi.nlm.nih.gov/)

α-d-glucose	β-d-glucose	α-d-galactose	α-d-mannose
500 mg/mL	1200 mg/mL	683 mg/mL	713 mg/mL

Along the lines drawn just above, i.e., by examining hydrogen bonding down to the electronic structure level, we hope to be able to see and demonstrate differences between the four sugars in question that lead to an understanding of, among others, their different solubilities. Further, our extensive studies of the hydration shell of these monosaccharides, based on ab initio molecular dynamics simulations (AIMD, see, e.g., ref (20)), may provide information concerning why these molecules can recognize some specific patterns on a macromolecule. The main advantage of AIMD over classical computer simulations is that AIMD is able to take charge transfer and polarization effects that arise from sugar–water interactions into account.

## Computational Details

The initial particle configurations were generated from classical MD simulations using GROMACS software.^34^ For monosaccharide molecules the Charmm36 all-atom interaction potential^12^ and for water molecules the SPC/E^35^ explicit water model were used. Periodic boundary conditions were applied, and the box sizes were 1.52577 nm (α-d-glucose), 1.52640 nm (β-d-glucose), 1.51058 nm (α-d-galactose), and 1.51170 nm (α-d-mannose) containing one monosaccharide molecule and 99 water molecules for a total of 321 atoms in each case. The average temperature of the four systems during the NVT simulations was 320 K using the Berendsen thermostat.^36^ The Newtonian equations of motion were integrated via the leapfrog algorithm using a time step of 1 fs. The total run time was 300 ns. The particle-mesh Ewald^37,38^ algorithm was used for handling the long-range electrostatic forces and potentials.

The present results arise from DFT-based (BLYP/D3) ab initio molecular dynamics simulations that have been performed at 320 K in a periodic setup using the CP2K^39^ ab initio molecular dynamics code. The norm-conserving Goedeker–Teter–Hutter pseudopotentials were used. We employed a triple-ζ basis set with double polarization (TZV2P). The plane-wave basis sets used a charge density cutoff of 300 Ry in the CP2K program. The time step was 0.5 fs. Particle configurations were collected for subsequent analyses from 100 ps runs after 40 ps of equilibration.

The Wannier centers (necessary for the molecule dipole moment calculations, see, e.g., ref (34)) were collected in every 50th time step (25 fs) in the trajectories of the production run. Fractional atomic charges were derived from the Bader-type analysis using Henkelman’s code^40^ and analyzed every 100th time step (every 50 fs).

Following our earlier work,^41^ two molecules were considered to be hydrogen-bonded to each other when they were at a distance r(O···H) < 2.5 Å and the H–O···O angle was <30°. The limiting distance in the definition is based on the well-defined first intermolecular minima of the corresponding partial radial distribution functions between the sugar and water hydroxyl groups. (cf. Figure S4). On the other hand, there are nearby water molecules that do not bond through H-bonds to the central sugar molecule but they form a van der Waals complex with the sugar molecule. Water molecules for which the condition r(C*_i_*···O_water_) < 4.5 Å is satisfied (c.f. Figure S5) are considered members of the hydrophobic shell. It is important to note that almost all H-bonded water molecules also fulfill this criterion, but for them, the rather strict angular constraint (see above) must also be satisfied.

## Results and Discussion

### Average Coordination Numbers

The total average coordination numbers can be taken as the sum of the numbers of hydrophilic (NHPHILE) and hydrophobic (NHPHOBE) components. The hydrophilic part incorporates all water molecules that are bonded to a central sugar molecule through a H-bond. Results from the present AIMD simulations, provided in Table 2, show that β-d-glucose molecules form the most H-bonds with the surrounding water molecules, while in the other three studied cases, the number of H-bonds differs only a little.

**Table 2:** Average Coordination Numbers for the Four Hexopyranose Monosaccharides Investigated Here (from the CP2K Simulation)

	α-d-glucose	β-d-glucose	α-d-galactose	α-d-mannose
NHPHILE	10.8	11.4	10.7	10.9
NHPHOBE	9.2	8.5	9.4	9.2

Concerning the hydrophobic shell, water molecules for which r(C*_i_*···O_water_) < 4.5 Å are taken into account. It is β-d-glucose that coordinates the smallest number of water molecules by hydrophobic interactions (see Table 2), whereas roughly the same number of non-hydrogen-bonded water molecules can be found in the first hydration shell of the other three sugar molecules. As far as we are aware, this property has not been investigated for these systems previously. It should be noted that the accuracy of the values in Table 2 (calculated using the bootstrapping method)^42,43^ for both the hydrophilic and hydrophobic hydration spheres was found to be within a few hundredths.

### H-Bonding Properties

**Figure 2:**
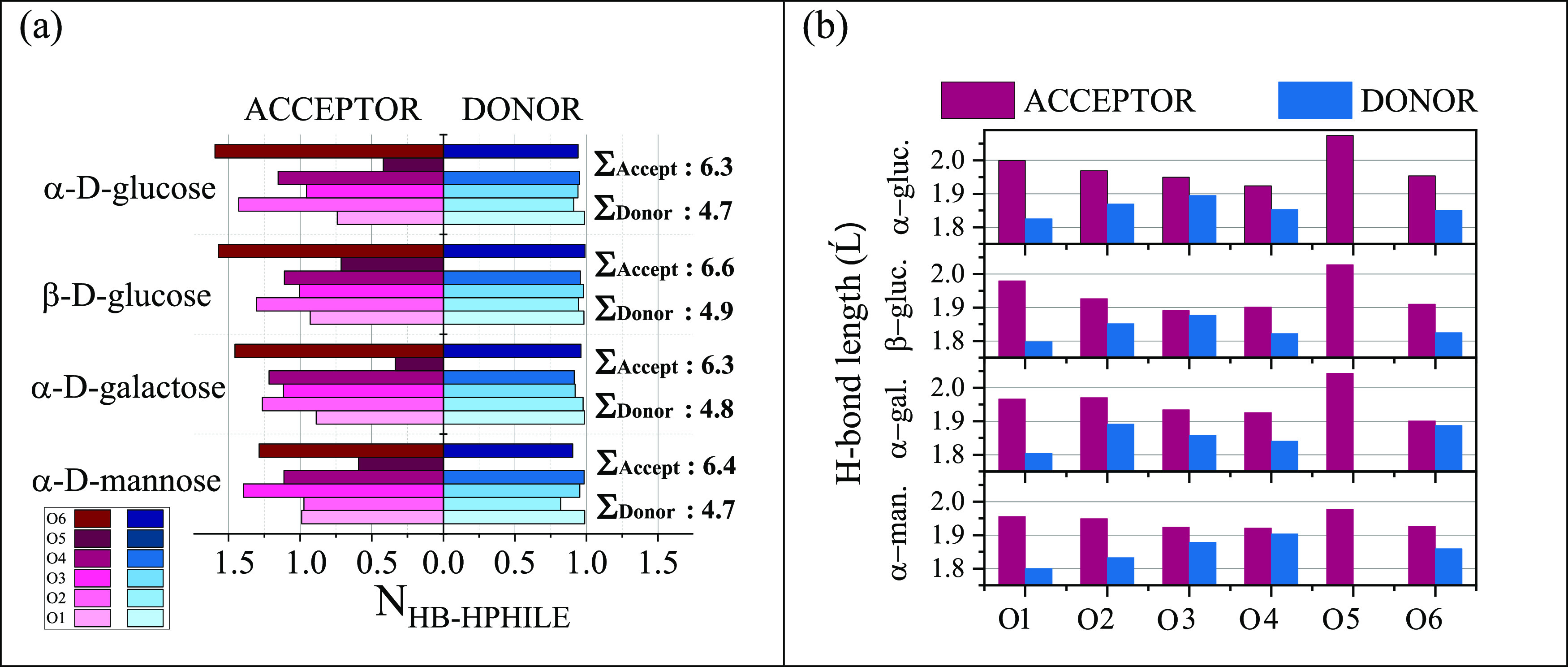
(a) Average number of acceptor and donor H-bonds. (b) Average length of acceptor and donor H-bonds.

The hydrogen-bonding ability of the different oxygen sites (for the assignation, c.f. Figure 1) is shown in Figure 2a. There are five hydroxyl groups in the monosaccharide molecules studied here, and all of them can act as donors of one H-bond and as acceptors of two H-bonds. Additionally, the ring O atom can form two H-bonds with water, as an acceptor. In all cases, the donor coordination number is larger than 0.9, but it stays below 1: these values are only slightly different among the sugar molecules considered here. The smallest average coordination number (approximately 0.81) belongs to the O2 donor site of α-d-mannose, which is somewhat out of line. Concerning acceptor properties, the O6 sites were found to be the most attractive, and the O2 site is the second most occupied. Again, α-d-mannose exhibits a slightly different behavior, as the best acceptor was found at the O3 site, and the second best is O6. For each studied case, the ring oxygens have proven to be the poorest acceptors.

Overall, β-d-glucose forms the highest number of H-bonds both as an acceptor and as a donor (see Figure 2). Another significant difference between the H-bonding abilities of different sugar molecules is the extent of involvement in H-bonding as an acceptor (see Figure 2a). For each of the sugar molecules we have studied, the O5 oxygen atom has the fewest water molecules attached to it by H-bonds. One finds the largest number of water molecules that H-bonds to the O5 position in β-d-glucose: this, in terms of H-bonding properties, is arguably the most significant difference between the monosaccharides studied here. Note also that the number of hydrogen bonds formed by sugars as donors and acceptors is slightly greater than the number of water molecules present in the first hydrophilic sphere, suggesting that some water molecules form H-bonds with sugars as both donors and acceptors (see Table 2 vs Figure 2a).

The strengths of the donor and acceptor types of H-bonds can be estimated by scrutinizing H*_i_*···O_water_ and O*_i_*···H_water_ distances (Figure 2b), where H*_i_* and O*_i_* are the hydrogen and oxygen atoms, respectively, of the hydroxyl group connected to the *i*th C atoms (c.f. Figure 1). It is found that the donor distances are significantly smaller than the acceptor distances: this implies that H-bond donor interactions are stronger than the acceptor ones. The longest average H-bond acceptor distance was detected for the O5···H_water_O-type H-bond in α-d-glucose. The largest differences between donor and acceptor H-bonded distances have always been detected at the O1 sites (Figure 2b). The shortest average donor H-bond length of a sugar molecule was revealed in β-d-glucose, while the longest one, related to the weakest H-bond strength, was found in α-d-glucose. In agreement with this, the average H-bond length was also the shortest in the β-d-glucose solution. More detailed, two-dimensional H-bond analyses also prove (Figures S7–S9) that a H-bond is more linear and shorter when the hydroxyl oxygen of the sugar molecule is a H-donor than when the same atom is a H-acceptor.

We note that Suzuki et al.,^21^ using a different approach, namely, the Wannier projection method,^42^ made a similar observation. They established that the H*_i_*···O_water_ (sugar–water) bond is shorter and the O*_i_*···H_water_ (sugar–water) bond is longer than the same type of H-bond between two water molecules in the bulk. Beyond the calculation of related characteristic distances, we also show in the Supporting Information that water–water interactions are highly dependent on the number of hydrogen-bonded neighboring molecules (c.f. Figure S6), i.e., on the coordination numbers (c.f. Table 2 and Figure 2a).

### Electronic Properties

**Figure 3:**
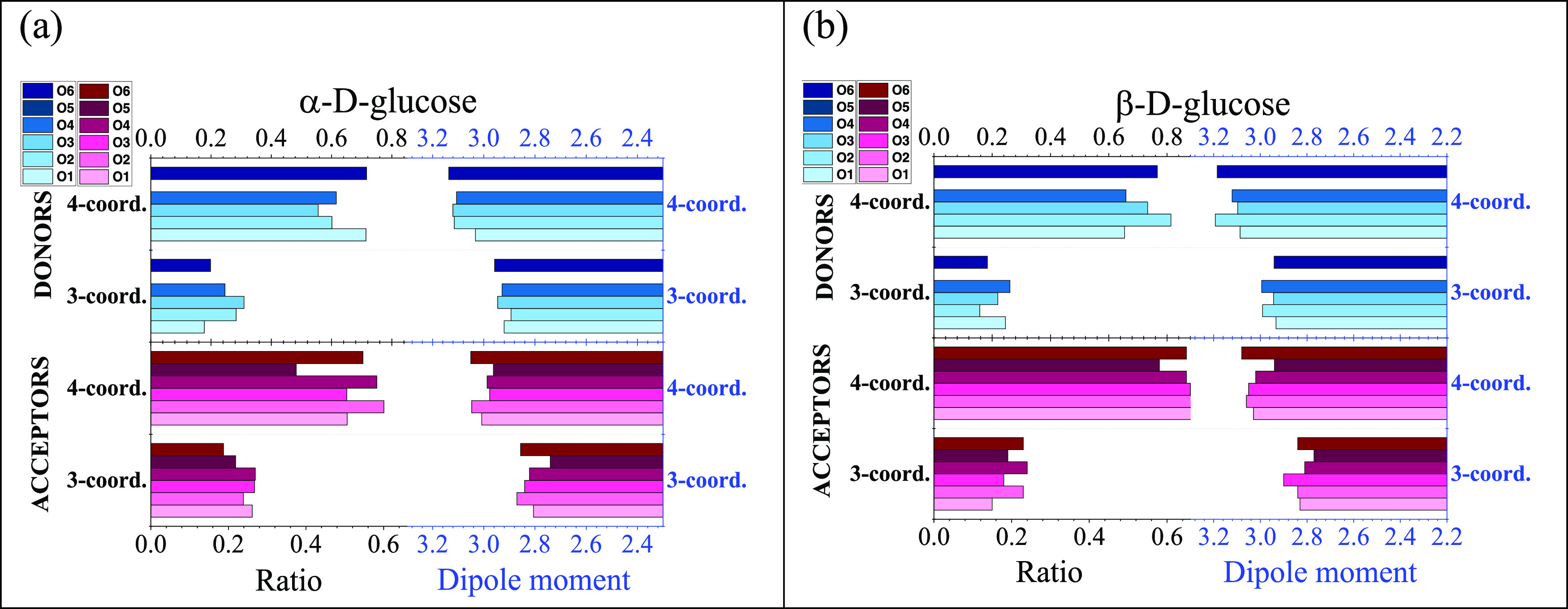
(a) Properties of three- and four-coordinated water molecules around α-d-glucose. (b) Properties of three- and four-coordinated water molecules around β-d-glucose.

Concerning electronic properties, the Wannier dipole moments of water molecules that are H-bonded directly to a monosaccharide are displayed in Figure 3: results are classified according to the H-bonded coordination number of water molecules. Water molecules, regardless of which sugar oxygen atom they are connected to, mostly are 4-fold-coordinated (one neighbor is the sugar and the other three are water molecules). It is found that the dipole moment of 4-fold-coordinated water molecules is larger than that of 3-fold-coordinated ones: this is the same behavior as found for molecules in pure water.^44^ That is, the dipole moment of water molecules, again (cf. also ref (44).), clearly depends on their H-bonded neighborhood.

Furthermore, the acceptor or donor nature of the monosaccharide molecules was also taken into account during this classification. Comparing the two glucose systems, no clear difference can be detected in terms of the behavior of “contact” water molecules that are located in the first shell of glucose molecules.

An interesting observation, however, is in order here: water molecules acting as (H−) donors (to the saccharide molecule) possess, on average, dipole moments larger than those with acceptor roles. Although this point, not being vital from the point of view of the main focus of the present work, is not pursued any further, the conjecture is worth making that this behavior occurs in parallel (or perhaps, even as a consequence) with that donor H-bonds are shorter (and thus, stronger) than acceptor ones (cf. Figure 2).

Turning our attention to the electronic properties of the monosaccharide molecules, Bader charges^45^ of OH groups and O5 atoms in the ring have been calculated. As seen in Table 3, the charges belonging to O2H, O3H, O4H, and O6H are almost identical for each investigated sugar. On the other hand, charges on the anomeric O1H groups are significantly different: they are the most negative, and among the sugars here, β-d-glucose sticks out with the largest negative value.

**Table 3:** Bader Charges /-*e*/ on the OH Sites of Sugar Molecules in the Solutions

systems	O1H	O2H	O3H	O4H	O6H
α-d-glucose	–0.669	–0.539	–0.544	–0.564	–0.551
β-d-glucose	–0.727	–0.568	–0.573	–0.570	–0.564
α-d-galactose	–0.625	–0.546	–0.567	–0.547	–0.547
α-d-mannose	–0.650	–0.554	–0.558	–0.565	–0.551

A similar conclusion can be drawn if charges on the O atoms in the sugar molecules are considered. The charge distribution on the O atoms is also the most pronounced on the O1 atom, as shown by the dipole moment data in Table 4. The smallest negative charge is on the O5 atom, which is clearly related to the fact that the O5···H_water_ H-bond is always the longest. Additionally, the total charge of each monosaccharide studied here in aqueous solutions is slightly negative: −0.022*e* (α-d-glucose), −0.018*e* (β-d-glucose), and −0.017*e* (α-d-galactose and α-d-mannose). This small negative charge is the result of a rather complex electron transfer process since sugar forms H-bonds with more water molecules as an acceptor than as a donor. Accordingly, sugar molecules should possess a positive charge. However, the length of the H-bonds is significantly shorter when the sugar molecule acts as a donor. During the formation of stronger H-bonds, there is a greater electron transfer from water molecules to sugar molecules than vice versa. (Note that this effect would not be visible in classical simulations.)

**Table 4:** Bader Charges /-*e*/ on the O Atoms in the Ring of Sugar Molecules in the Solutions

systems	O1	O2	O3	O4	O5	O6
α-d-glucose	–1.316	–1.206	–1.197	–1.232	–1.054	–1.209
β-d-glucose	–1.407	–1.230	–1.243	–1.237	–1.064	–1.220
α-d-galactose	–1.273	–1.200	–1.222	–1.203	–1.067	–1.200
α-d-mannose	–1.324	–1.207	–1.216	–1.213	–1.104	–1.200

The magnitude of the charge (Table 4) and the dipole moment (Table 5) connected to the O atoms characterize the asymmetry of the charge distribution within the monosaccharide molecules: both the charge and the dipole moment are always the largest for the O1 oxygen. Furthermore, both values are the highest for β-d-glucose among the monosaccharides studied here.

**Table 5:** Dipole Moment /D/ of the O Atoms in the Ring of Sugar Molecules in the Solutions

systems	O1	O2	O3	O4	O5	O6
α-d-glucose	0.507	0.391	0.373	0.411	0.240	0.386
β-d-glucose	0.625	0.389	0.433	0.405	0.247	0.378
α-d-galactose	0.462	0.389	0.416	0.367	0.265	0.382
α-d-mannose	0.525	0.395	0.390	0.368	0.312	0.377

### Hydration Sphere of Monosaccharides

**Figure 4:**
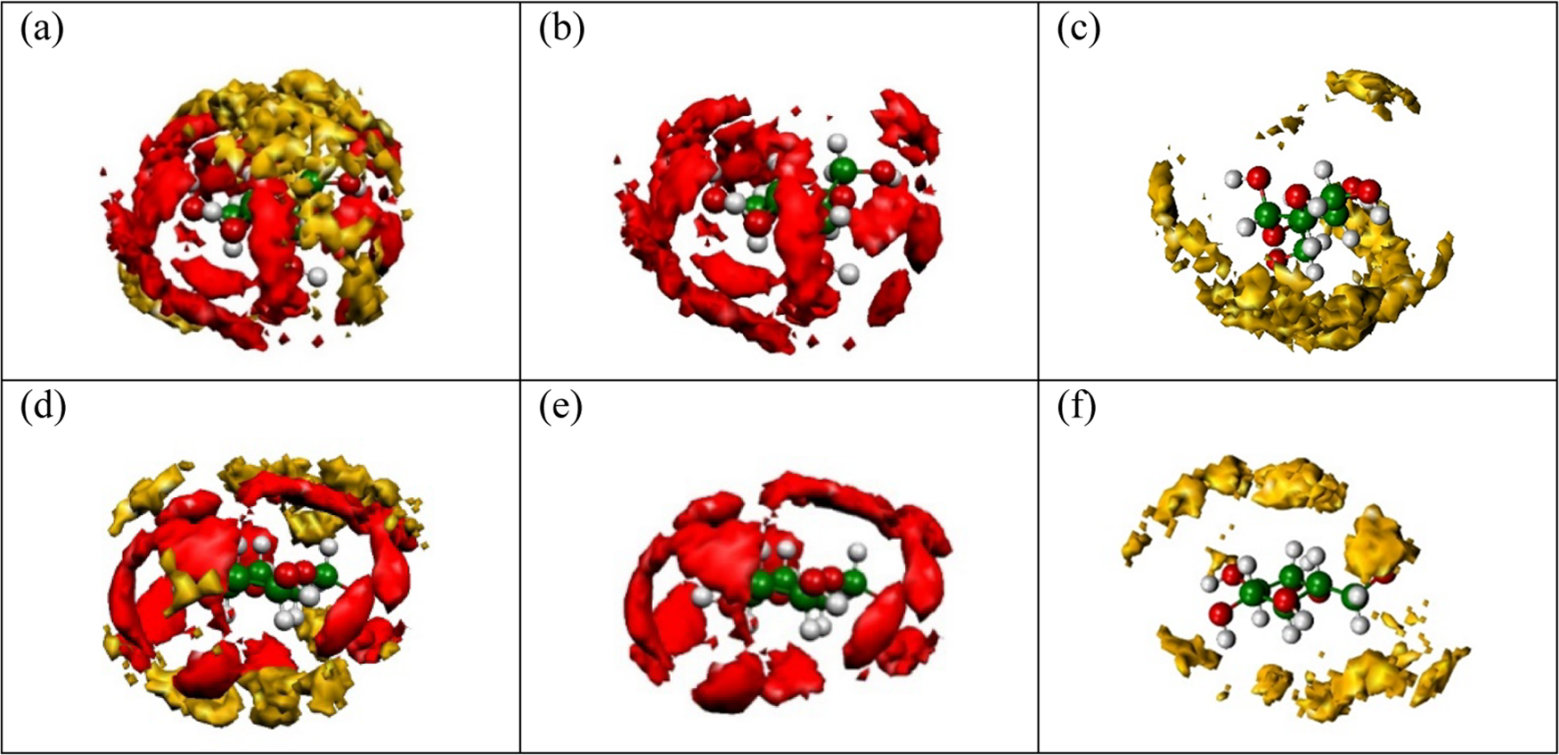
Spatial distribution functions around α-d-glucose (top panels) and β-d-glucose (lower panels). (a, d) Hydrophobic and hydrophilic shells together. (b, e) Hydrophilic shell only. (c, f) Hydrophobic shell only.

Spatial distribution functions^46^ (SDFs) around central sugar molecules have also been calculated. SDFs are able to provide unique three-dimensional (3D) representations of hydration shells (hydrophilic and hydrophobic) around a central monosaccharide molecule that are the consequence of the different orientations of hydroxyl groups. The origin of the coordinate system was the center of mass (CM) of the sugar; the XY plane is defined by the positions of C5, CM, and C1; and the + *x* direction is defined as the bisector of the C5—CM—C1 angle. Figure 4a–c displays the SDF for α-d-glucose and Figure 4d–f for β-d-glucose, as typical examples.

The red and yellow colors denote the hydrophilic and hydrophobic shells, respectively. Note that the average density of O_water_ atoms around a monosaccharide molecule is higher than the bulk density of water (0.033 molecules/Å^3^). Water molecules are located in well-identifiable regions around the sugar: in the directions of hydroxyl groups (along the O_sugar_–H_sugar_ vector) and the lone pairs of O_sugar_. In hydrophobic shells, these regions are mainly located in the direction of C_sugar_–H_sugar_.

**Figure 5:**
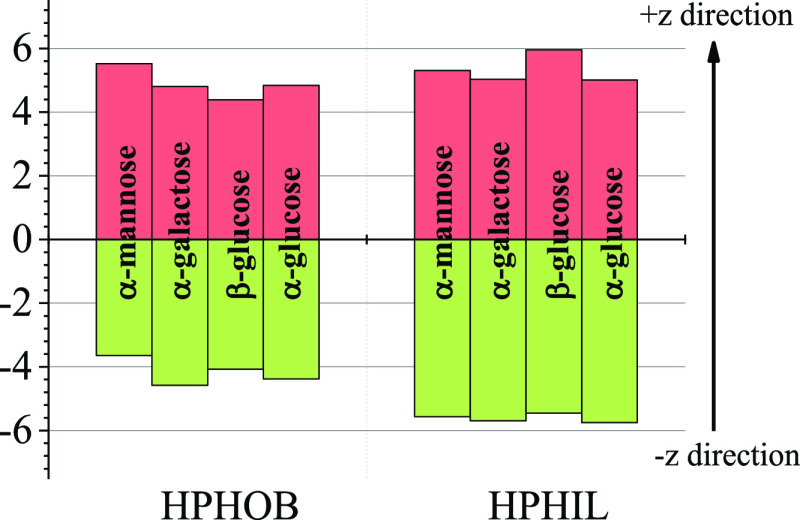
Number of hydrophobic and hydrophilic H-bonded water molecules above and below the XY molecular plane of monosaccharide molecules considered here.

In order to extract quantitative information from the SDF, we determined the number of water molecules below and above the XY plane of the sugar molecules for both types of hydration spheres (Figure 5). In general, there is a significantly larger number of water molecules in the hydrophobic shell above the plane: β-d-glucose is one of the most symmetric in this respect. On the other hand, the hydrophilic hydration sphere of each, but of β-d-glucose, monosaccharide contains significantly more molecules below the plane. This is related to the direction of the OH group (O1) attached to the first carbon atom (C1): for α anomers, the −CH_2_OH (C6 and O6) group lies on the opposite side of the XY plane of the molecule, while for the β anomer, O1 and O6 are on the same side (cf. Figure 1). It may be conjectured then that these asymmetric features of the hydration shell can play a significant role in molecular recognition. It should be noted that the accuracy of the values in Figure 5 (calculated, again, using the bootstrapping method^42,43^) was found to be within a few hundredths.

## Conclusions

In summary, ab initio molecular dynamics simulations have been performed for aqueous solutions of four simple sugars, α-d-glucose, β-d-glucose, α-d-mannose, and α-d-galactose.

(1) It has been established that β-d-glucose forms the highest, while α-d-glucose forms one of the smallest, number of hydrophilic hydrogen bonds with surrounding water molecules. Concerning hydrophobic interactions with nearby water molecules, β-d-glucose has the smallest number of water bounded hydrophobically. These findings are in accord with the fact that β-d-glucose is the most and α-d-glucose is the least soluble in water.
(2) The largest number of water molecules that H-bonds to the O5 position is found in the aqueous solution of β-d-glucose.
(3) The average H-bond length when the OH group of the sugar molecule is involved as a donor in H-bonds was the shortest/longest for β-d-glucose/α-d-glucose, respectively. In agreement with this, the average H-bond length was also the shortest for β-d-glucose.
(4) The Bader charges derived from AIMD simulations for oxygen atoms at positions 2, 3, 4, and 6 are very similar for all monosaccharides studied. On the other hand, the O atom at position 1 has a significantly more negative charge. Of all of the O atoms, the one at position 5 has the least negative charge.
(5) Scrutinizing the spatial structure of the hydration spheres of sugar molecules has revealed apparent asymmetries of the hydrophobic shell, whereas the hydrophilic shell is much more symmetric. β-d-glucose appears to be outstanding in terms of both of its hydrophilic and hydrophobic hydration shells.

## Supporting Information Available

Self-diffusion constants calculated by the Einstein relation, without taking PBC effects into account (Table S1); characteristic time of reorientation of OH vectors of water molecules in the studied monosaccharide solutions (Table S2); lifetimes of H-bonds (Table S3); H-bond number distributions of water–water and sugar–water pairs (Figure S1); average H-bond numbers (Table S4); characteristic dihedral angles (Figure S2); two-dimensional contour plots of C2–C1–O1–H1 and O6–C5–C5-O5 dihedral angles (Figure S3); H*_i_*–O_water_ (*i* = 1,2,3,4,5) partial radial distribution functions for β-d-glucose (Figure S4a); O*_i_*–H_water_ (*i* = 1,2,3,4,5,6) partial radial distribution functions for β-d-glucose (Figure S4b); C*_i_*–O_water_ partial radial distribution functions (Figure S5); characteristic distances and angles related to the centers of localized orbitals of O atoms (of water, monosaccharide) (Figure S6); two-dimensional distribution functions characterizing the H-bond interactions for α-d-glucose (Figure S7); two-dimensional distribution functions characterizing the H-bond interactions for β-d-glucose (Figure S8); differences of the 2D joint probability of donor and acceptor sites of the O1 atom (Figure 9a); differences of the 2D joint probability of the O1 site of α- and β-d-glucose (Figure 9b); average H-bond numbers (Table S5); average H-bond distances (Å) (Table S6); average dipole moment (D) of water as a function of H-bonded neighbor numbers (Figure S10); and average dipole moment (D) of water connected to the OH and ring O (Table S7) (PDF)
