# Supplementary material for: Outstanding Properties of the Hydration Shell around β-d-Glucose: A Computational Study
Source: ACS Omega. 2024 Apr 25;9(18):20331–7. doi: 10.1021/acsomega.4c00798 (PMC11080014; doi:10.1021/acsomega.4c00798)
Supplement: Supplementary file 1 — ao4c00798_si_001.pdf [file ao4c00798_si_001.pdf]

Supporting Information for

Outstanding Properties of the Hydration Shell Around β-D-Glucose: A Computational Study

Imre Bakó[a], László Pusztai[b,c,] Szilvia Pothoczki[b*]

[a] HUN-REN Research Centre for Natural Sciences, H-1117 Budapest, Magyar tudósok körútja 2., Hungary

[b] HUN-REN Wigner Research Centre for Physics, H-1121 Budapest, Konkoly-Thege M. út 29-33., Hungary

[c] International Research Organization for Advanced Science and Technology (IROAST), Kumamoto University, 2-39-1 Kurokami, Chuo-ku, Kumamoto, 860-8555, Japan

*Correspondence to: pothoczki.szilvia@wigner.hun-ren.hu

Table of Contents:

1. Dynamical properties

    1A. Self-diffusion coefficients and reorientational time

    1B. Hydrogen-bond lifetime

2. Conformational analyses

3. Partial radial distribution functions

4. Cooperative behavior of water molecules around the sugar molecule

5. Two-dimensional distribution functions characterizing the H-bond interactions

6. Structural criteria for hydrogen bonds

7. Dipole moment of water molecules

8. Comparison between the experimental and theoretical hydration numbers of monosaccharides

1. Dynamical properties

A. Self-diffusion coefficients and re-orientational time

The self-diffusion coefficient (D) was calculated using the Einstein-Smoluchowski relation from the mean squared displacements of the O atoms of water molecules:

$$D = \lim_{t\to\infty} \frac{1}{6Nt}\left\langle \sum_{i=1}^{N}\left(r_i(t) - r_i(0)\right)^2 \right\rangle \quad (1)$$

where $r_i(t)$ and $r_i(0)$ are the positions of the centres of O atoms of water molecules at time t and 0, respectively, and the <…> denotes an ensemble average.

The calculated self-diffusion coefficients for water (D_2O) in our investigated systems are presented in Table S1. In our previous liquid water AIMD simulations using the same type of parameters (functional, cutoff, time step)[1], the self-diffusion coefficient of D_2O was 0.16 ± 0.03 at 300 K and 350 K and 0.31 ± 0.03 Å2/s at 300 K. These calculated values show that there is no supercooled liquid state in any of the systems we have studied, but the real "experimental temperature" is smaller than 320 K. If we make a direct comparison with experimental diffusion constants for liquid D_2O[2], we can conclude that the measurements suggest that the real temperature is around 280 K.

Table S1. Self-diffusion constant calculated from the Einstein relation, without taking PBC effects into account.

Systems	Self-diffusion constant (10^{-8} m^2/s)
α-D-glucose	0.125
β-D-glucose	0.117
α-D-galactose	0.101
α-D-mannose	0.127
water	0.160(1)

It has already been shown[3-6] that the O-H reorientational dynamics are significantly perturbed by the local environment (H-bonded environment) around a central molecule. These quantities related to the orientational relaxation of the O–H vectors can be measured by ^1H–^{17}O dipolar relaxation NMR experiments. Experimentally, the corresponding value is in the range of 1.7-2.6 ps but significantly decreases with the temperature[2,7-10]. For example, in the case of TIP5P water model, this value is about 2ps at 300 K and 6 ps at 280 K.[3]

Reorientational dynamics have been characterized by the autocorrelation functions:

$$C_l(t) = \langle P_l(\underline{e}(t)\cdot \underline{e}(0)\,)\rangle \quad (2)$$

where $\underline{e}(t)$ is the unit vector along a well-defined molecular axis (O-H) vector, perpendicular to the HOH water molecular plane, and P_l is the l-th Legendre polynomial.

The decay time of reorientational autocorrelation functions, <τ>, is estimated by computing the integral of $C_l(t)$ in terms of time, that is:

$$\tau = \int_0^{\infty} C_l\,(t)dt \quad (3)$$

It is well established that the $C_l(t)$ functions do not show an exponential decay at short times the integrals were carried out numerically up to 7 ps. The contribution from the long tail part of $C_l(t)$ functions was calculated using a fitting procedure, and the integral was carried out based on the fitting value of exponential decay. Our data show that the real temperature is smaller than 320 K. The calculated reorientational times are presented in Table S2.

Table S2. The characteristic time of reorientation of OH vectors of water molecules in the studied monosaccharide solutions (in ps).

	P1(OH) (ps)	P2(OH) (ps)	P HOH plane (ps)
α-D-glucose	8.07	4.39	6.25
β-D-glucose	11.11	6.58	8.93
α-D-galactose	9.44	6.25	7.15
α-D-mannose	10.42	5.44	6.58

B. Hydrogen-bond lifetime

The average hydrogen bond lifetime was calculated from the probability distribution of H-bond lifetimes. In this calculation, the formation/re-formation of H-bonds over a short period (0.1 ps) was taken into account. Note that the so-called transient H-bonds that exist only over a short period (0.1 ps) without re-formation later within a similar time interval have been excluded from our calculations.[11-20] This type of calculation for H-bond lifetime is called a history-dependent calculation. In these calculations, in addition to examining water-water and sugar-water H-bonds, we also calculated the lifetimes of bonds where the sugar molecule participates as an H-donor or H-acceptor. We found that the donor lifetimes are significantly longer than those calculated for the acceptor case in all cases studied. These results are in good agreement with the statement that the length of the H-bond in the donor case is significantly shorter than that in the acceptor case. The H-bond lifetimes were highest for β-D-glucose (see Table S3). Corresponding to this fact, the strongest H-bond interaction was identified between sugar and water molecules. The average H-bond lifetime of water-water is similar to the sugar-water H-bond lifetime, where sugar is the acceptor of the H-bond.

Table S3. Lifetimes of H-bonds (in ps).

Lifetime (ps)	HB (acceptor)	HB (donor)	HB (water-water)	HB (sugar-water)
α-D-glucose	2.04	5.05	1.79	2.89
β-D-glucose	3.33	8.23	2.18	4.57
α-D-galactose	2.09	5.61	1.97	3.20
α-D-mannose	1.81	3.06	2.00	2.30

We examined what happens if we take into account, and correct for, H-bond termination/formation over a short period (about 0.1 ps), in a similar way to that described by Clark et al.[11] The H-bond number distributions of water-water and sugar-water are shown in Figure S1. It can be seen that the correction due to the formation/reformation of the H-bonds (largely due to the arbitrariness of the definition) causes a well-defined change in the distribution functions. The average H-bond numbers (Table S4) vary slightly (about 2.5-4.0 %t) when this correction is taken into account. The magnitude of these corrections is similar to that described earlier by Clark et al.[11] Our results suggest that the inclusion of these corrections does not change our conclusions.

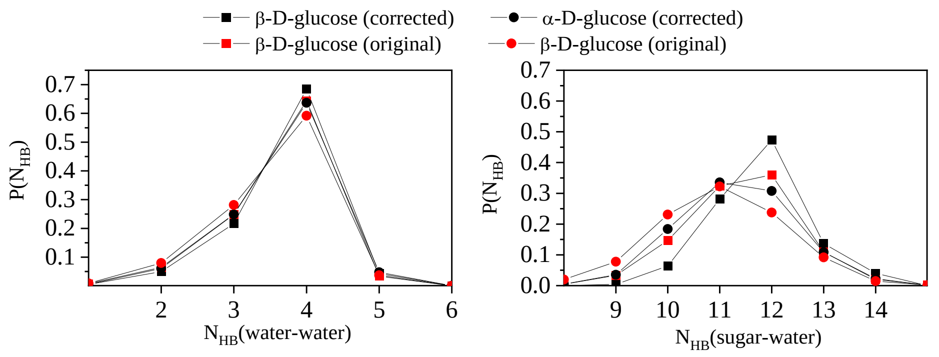

Figure S1. The H-bond number distributions of water-water and sugar-water pairs.

Table S4. The average H-bond numbers.

	β-D-glucose	α-D-glucose	α-D-mannose	α-D-galactose
sugar-water (corrected)	11.8	11.4	11.5	11.6
sugar-water (original)	11.4	10.8	10.7	10.9
water-water (corrected)	3.7	3.6	3.7	3.7
water-water (original)	3.6	3.5	3.6	3.6

2. Conformational analyses

Pyranose rings remained in their $^4C_1$ state during the simulations. A recent publication[21] revealed that the $^4C_1$ conformation is stable even in a very long simulation (5 μs). The authors showed that, according to Protein Data Bank, more than 80 % of the molecules are in $^4C_1$ configuration for the monosaccharides investigated here.[22] To present detailed conformational profile O5-C5-C6-O6, O5-C5-C6-O6 and C2-C1-O1-H1 dihedrals are shown in Figs. S2 and S3. In the classical MD simulations, the force field defined dihedral angle distributions were detected, with reasonable agreement with the experimentally determined abundance. In the AIMD simulations, α- and β-D glucose and α-D-galactose molecules, with respect to the hydroxymethyl group, are in the gauche-trans state, whereas about 17% of the saccharide molecules were found in the gauche-gauche conformation in the α-D-mannose solution.

Figures S2 and S3 show the conformation analyses in the classical MD (left-hand panels) and the ab initio MD simulations (right-hand panels) for the four studied monosaccharides. In the classical MD simulations, we reproduced the literature conformer ratio.[22] Note that in ab initio MD simulations the time scale does not allow for the sugar molecules to turn from one conformational state to another.

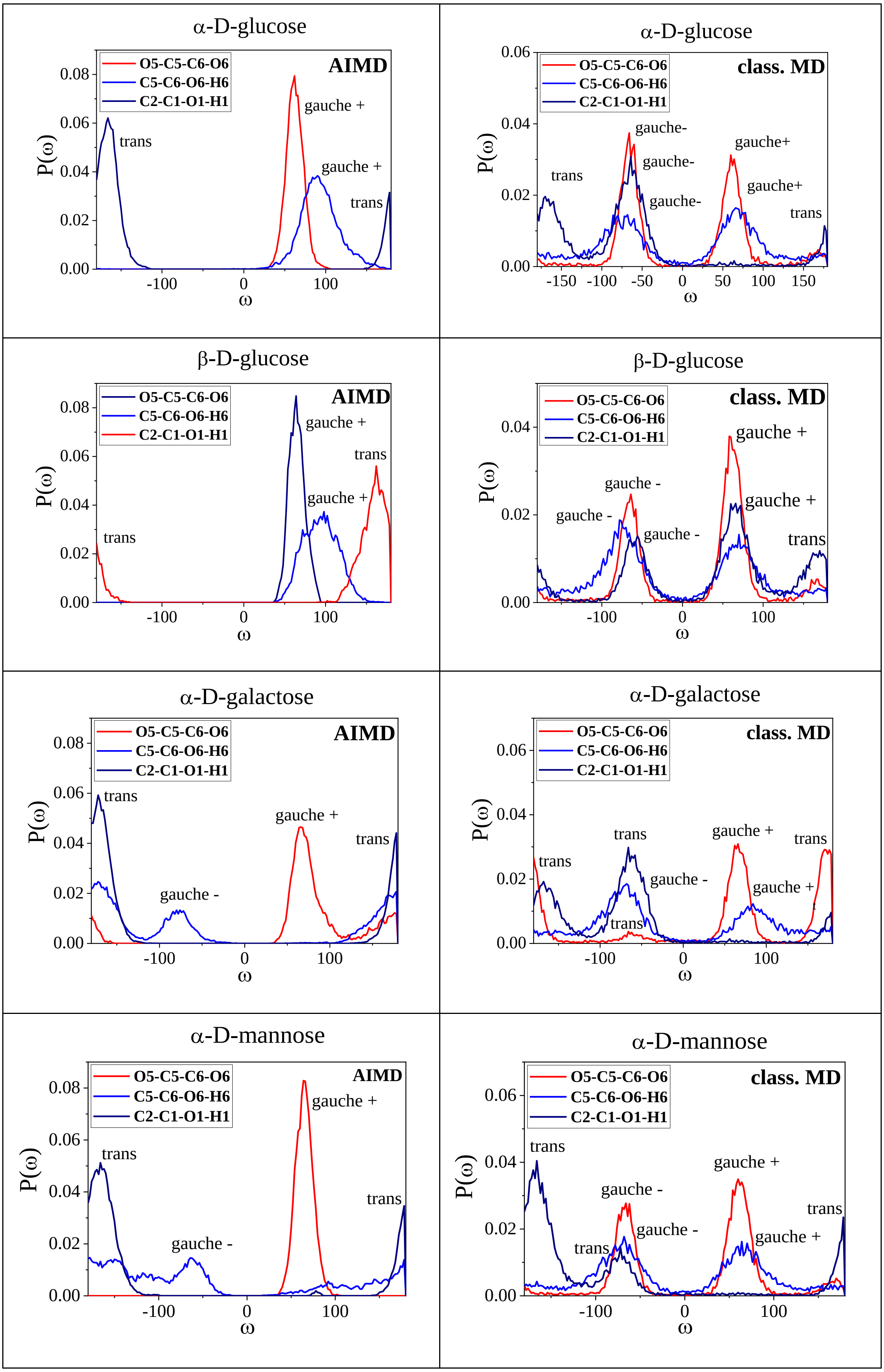

Figure S2. Characteristic dihedral angles.

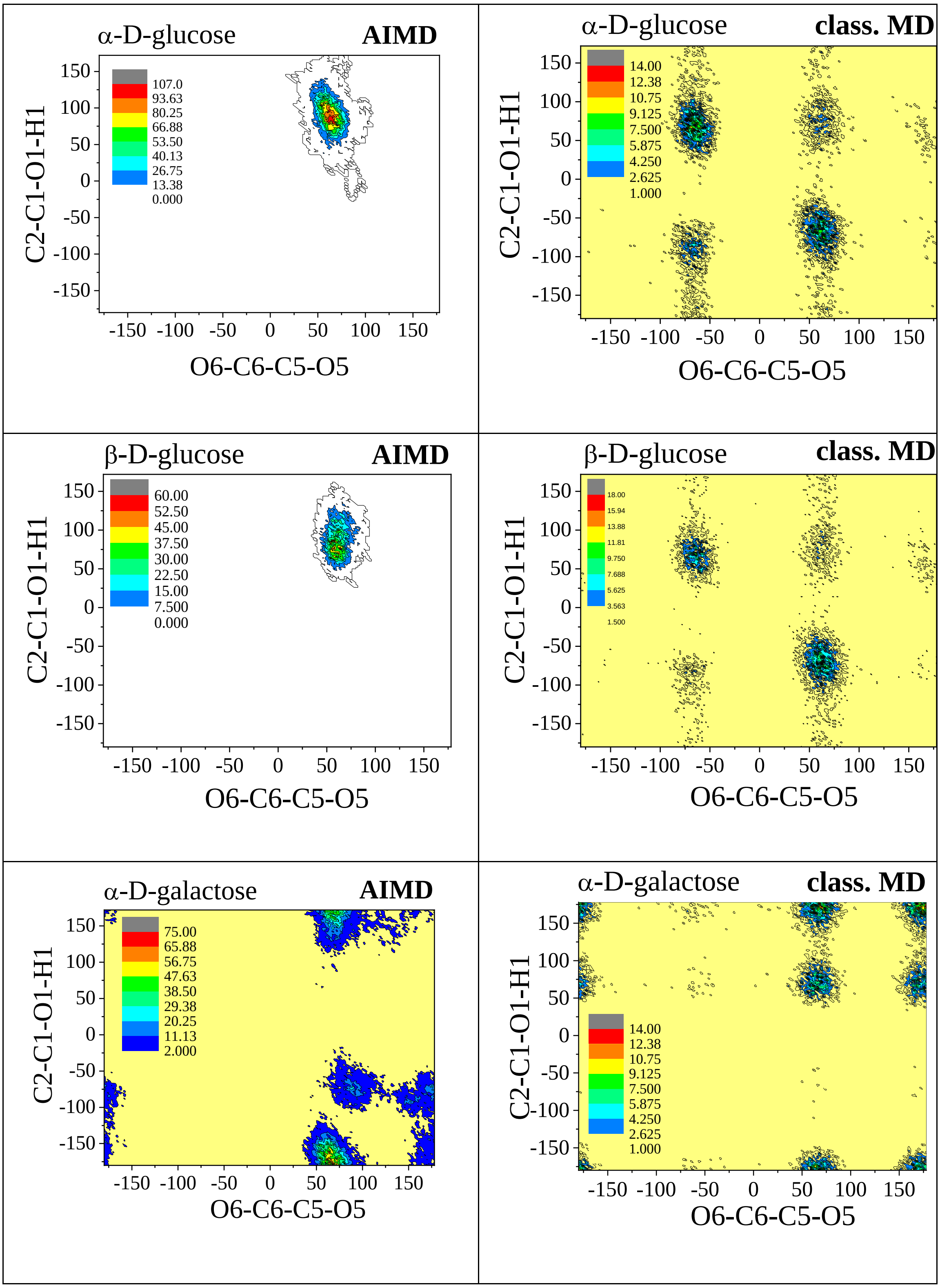
α-D-glucose
AIMD
107.0
93.63
80.25
66.88
53.50
40.13
26.75
13.38
0.000
C2-C1-O1-H1
O6-C6-C5-O5
α-D-glucose
class. MD
14.00
12.38
10.75
9.125
7.500
5.875
4.250
2.625
1.000
C2-C1-O1-H1
O6-C6-C5-O5
β-D-glucose
AIMD
60.00
52.50
45.00
37.50
30.00
22.50
15.00
7.500
0.000
C2-C1-O1-H1
O6-C6-C5-O5
β-D-glucose
class. MD
18.00
15.94
13.88
11.81
9.750
7.688
5.625
3.563
1.500
C2-C1-O1-H1
O6-C6-C5-O5
α-D-galactose
AIMD
75.00
65.88
56.75
47.63
38.50
29.38
20.25
11.13
2.000
C2-C1-O1-H1
O6-C6-C5-O5
α-D-galactose
class. MD
14.00
12.38
10.75
9.125
7.500
5.875
4.250
2.625
1.000
C2-C1-O1-H1
O6-C6-C5-O5

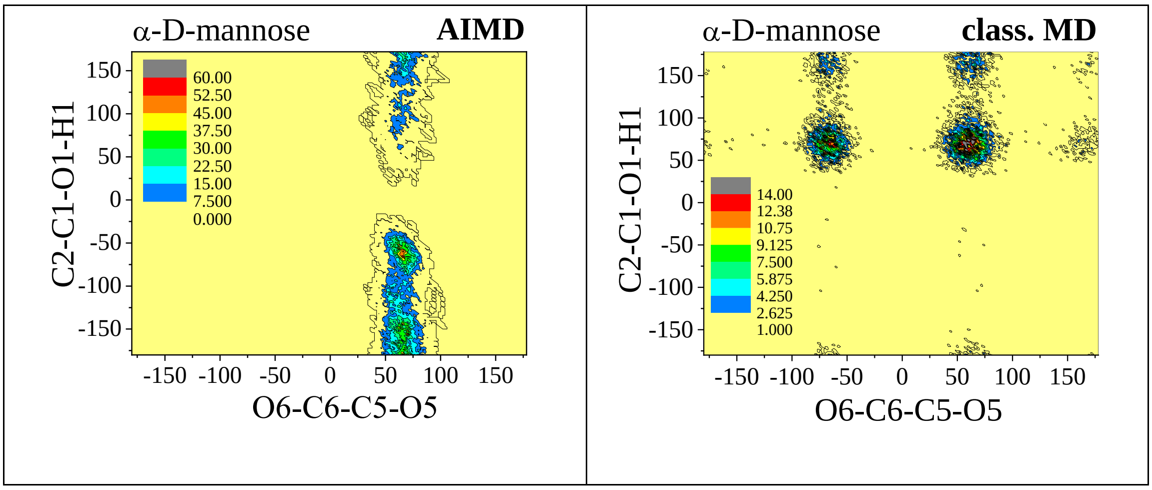

Figure S3. Two-dimensional contour plots of C2-C1-O1-H1 and O6-C5-C5-O5 dihedral angles.

3. <u>Partial radial distribution functions</u>

Figure S4 shows the partial radial distribution functions between the sugar and water hydroxyl groups, namely H_i-O_{water} (i=1,2,3,4,5) and O_i-H_{water} (i=1,2,3,4,5,6) for β-D-glucose.

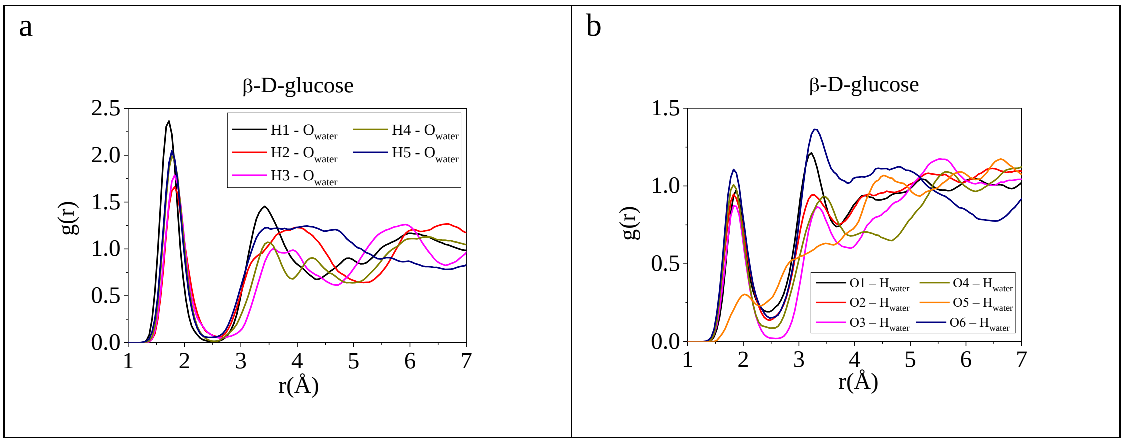

Figure S4. (a) The H_i-O_{water} (i=1,2,3,4,5) partial radial distribution functions for β-D-glucose. (b) The O_i-H_{water} (i=1,2,3,4,5,6) partial radial distribution functions for β-D-glucose.

The C_i-O_{water} (i=1, 2, 3, 4, 5, 6) radial distributions are presented in Figure S5 for β-D-glucose.

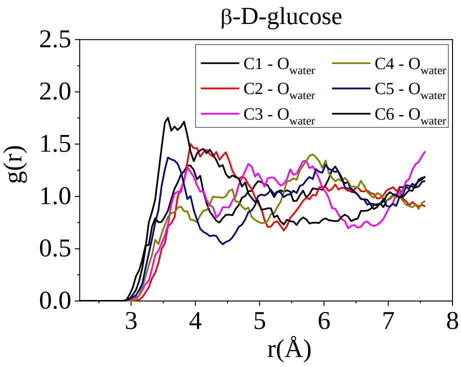

Figure S5. C_i-O_{water} partial radial distribution functions.

4. Cooperative behavior of water molecules around the sugar molecule

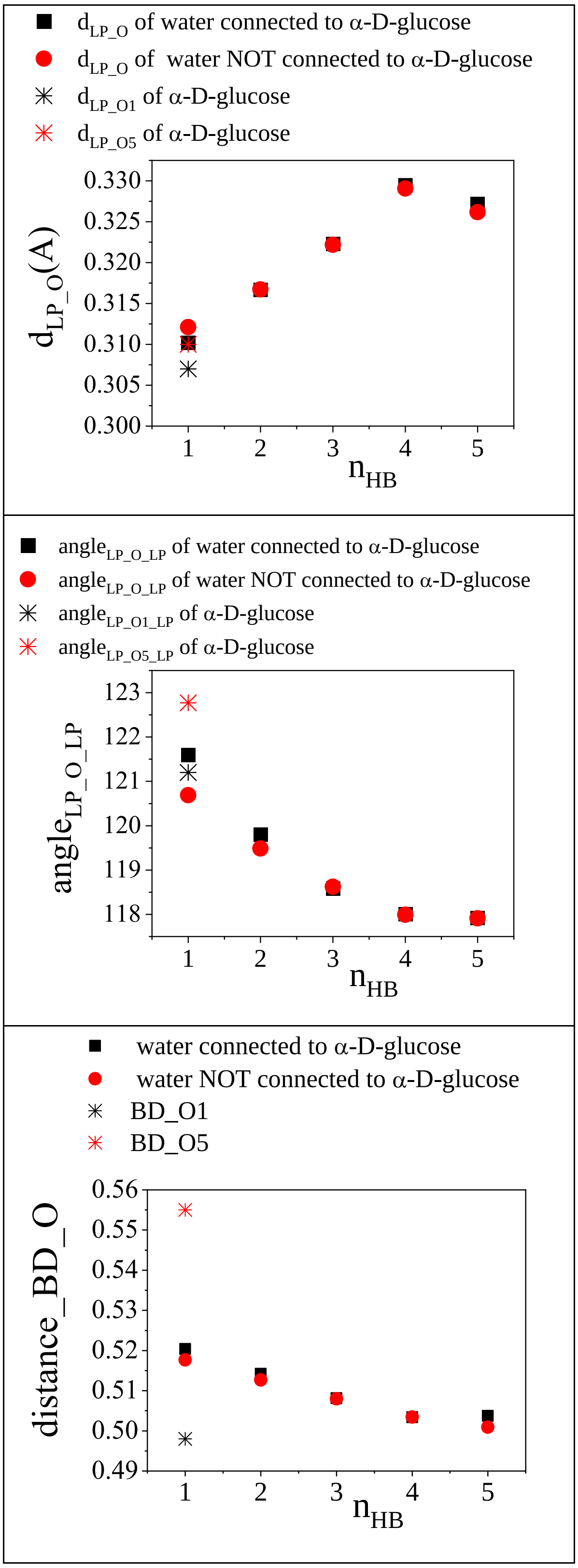

Figure S6. Characteristic distances and angles related to the centers of localized orbital of O atoms (of water, monosaccharide).

In water molecules, the four localized (hydrogen-bonding) orbitals lie in the directions of the two lone pairs of O atoms (LP_O), and of the two O-H vectors (BD_O). For monosaccharides, these localized orbitals can be found in the directions of the lone pairs of O_{sugar}. O_{sugar}-H and O_{sugar}-C_{sugar} directions. We calculated the average distance from the oxygen atoms to the centers of the lone pairs of O-atoms of water molecules (d_{LP_O}) that are, and also, those that are *not*, connected to monosaccharides. Fig. S6 shows the average distance from the O1 (d_{LP_O1}) and O5 (d_{LP_O5}) sites to the centres of the lone pairs for α-D-glucose. d_{LP_O} increases almost linearly with the number of hydrogen-bonded neighbours, which suggests an increasing interaction strength (since the lone pair gets a little closer to the neighboring atom). Similar behavior was detected in pure liquid water.[23] d_{LP_O1} and d_{LP_O5} (referring to O atoms in saccharide molecules) are shorter than the shortest d_{LP_O} d (O atoms in water molecules) distance. 'distance_BD_O' (in the bottom part of Fig. S6) refers to the distance between an O atom and the center of the (localized) electronic density in the direction of the covalently bonded neighbour (H in water, and H or C in the saccharide). Note that 'distance_BD_O' also varies systematically with the number of H-bonded neighbours.

5. Two-dimensional distribution functions characterizing the H-bond interactions

The strength of the hydrogen bond can also be measured by the joint angle of cos (H-O…O) and OH distance probability distribution. In the case of α-D-glucose and β-D-glucose this distribution is shown in Figs. S7 and S8 for hydroxyl groups of O2, O3, and O4 sites, as well as O1 site. We presented separately according to OH groups of monosaccharides formed H-bonds with water as H-bond acceptor and donor role.

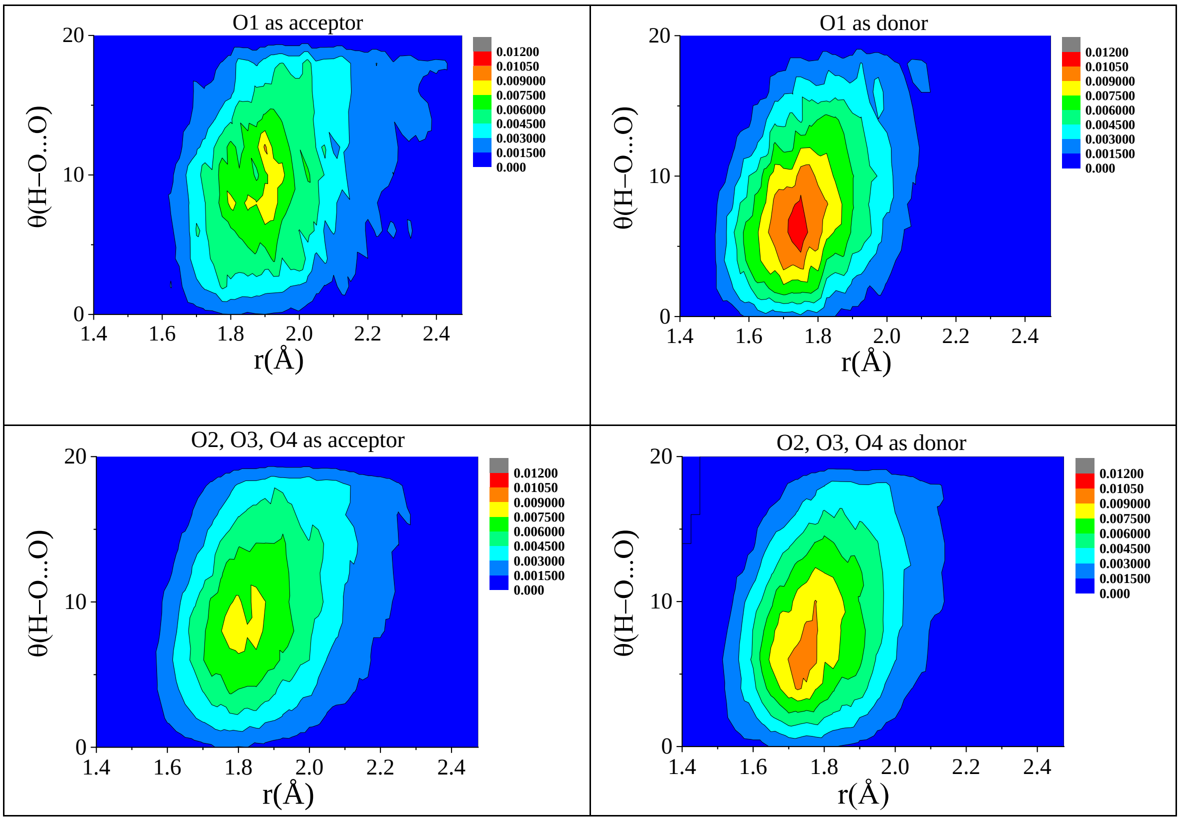

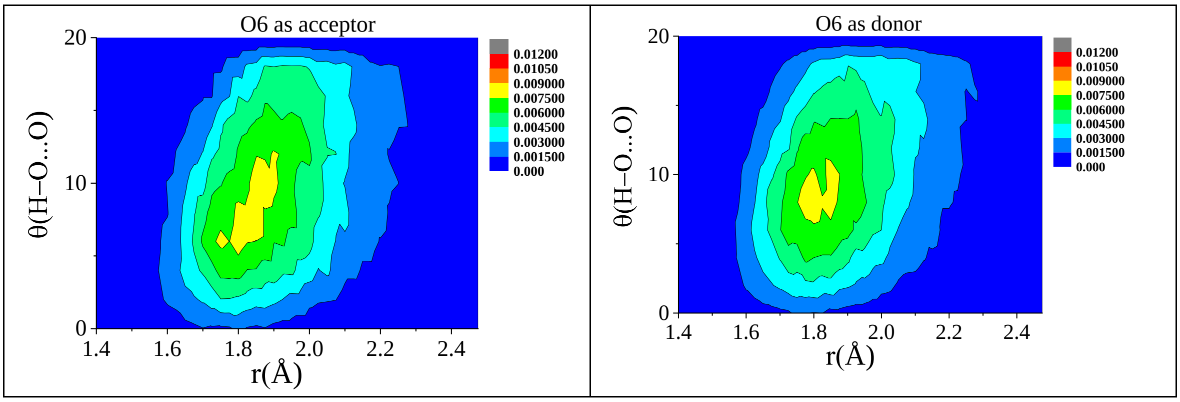

Figure S7. Two-dimensional distribution functions characterizing the H-bond interactions for α-D-glucose.

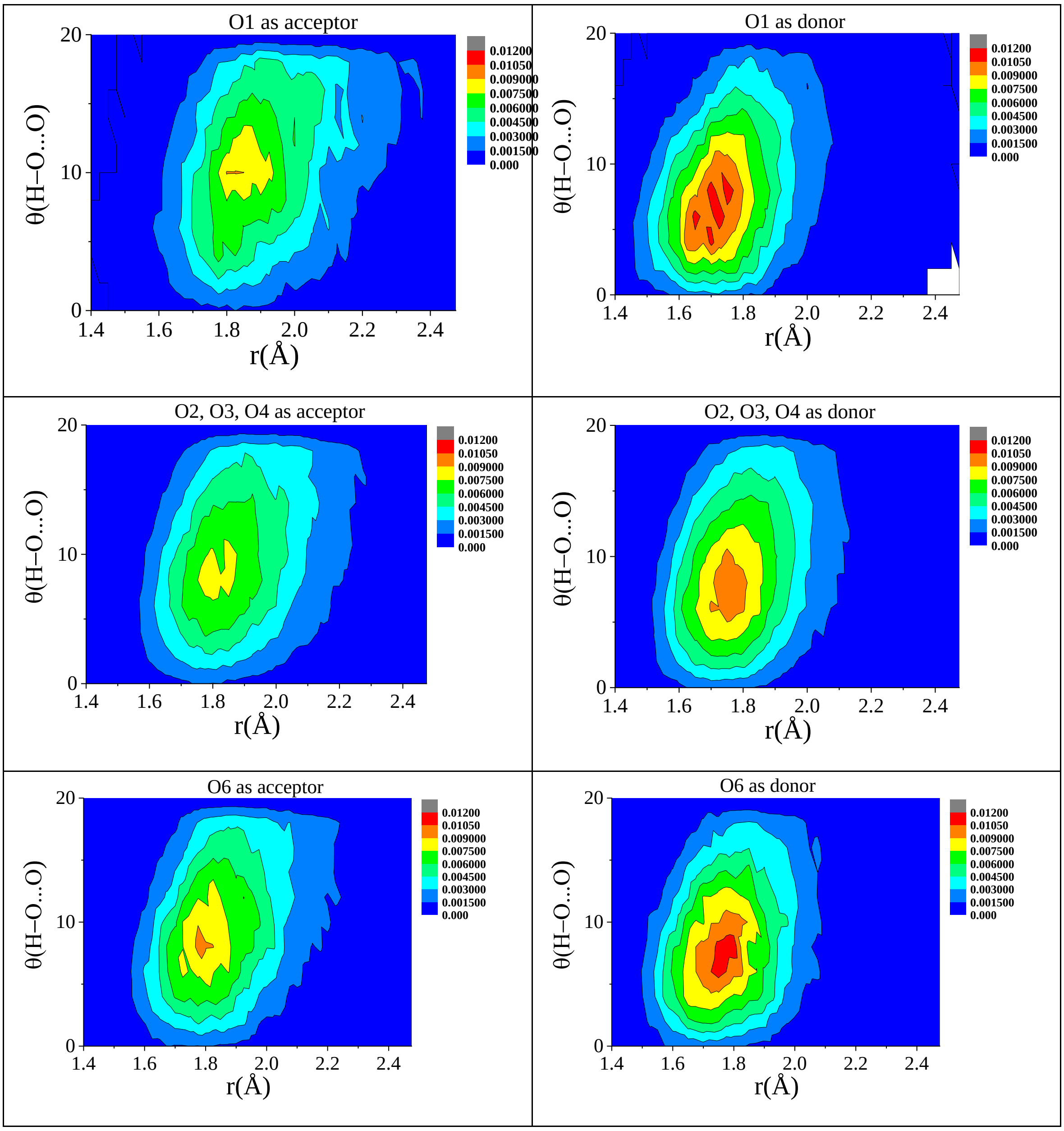

Figure S8. Two-dimensional distribution functions characterizing the H-bond interactions for β-D-glucose.

All joint probability distributions are normalized to unity. In this case, the following difference can be formed:

$$\Delta P(\theta, r_{OH}) = P(\theta, r)_{acceptor} - P(\theta, r)_{donor} \quad (4)$$

This difference in the case of the O1 oxygen atom is shown in Figure S9.a for α-D-glucose. From this figure, we can conclude that the donor type hydrogen bonding (H1-O1…O_w) occurs at significantly shorter distances and smaller angles than in the case of acceptor (O1...H_w-O_w). In Figure S9.b, we compare the 2D distribution functions defined above for the distributions for O1 as donor make a difference between β- and α- D-glucose. We found good agreement with the results obtained only for the average H-bond distance. We can conclude that the H-bond is shorter and more linear for β-D-glucose.

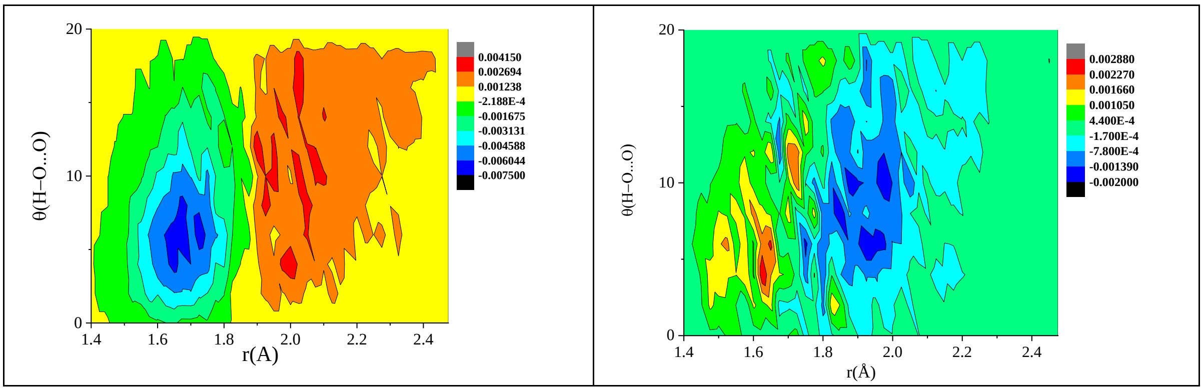

Figure S9. a) Differences of 2D joint probability of donor and acceptor sites of O1 atom b) Differences of 2D joint probability of O1 site of α- and β-D-glucose

6. <u>Structural criteria for hydrogen bonds</u>

In our calculations, two molecules were considered to be hydrogen bonded to each other when they were at a distance r(O···H) < 2.5 Å and the H-O…O angle was < 30°. We investigated to what extent our conclusions change if we change the definition r(O···H) < 2.5 Å and the H-O…O angle was < 20°. The average H-bond numbers and the average H-bonded distances are shown in Tables S5 and S6, respectively.

Table S5. The average H-bond numbers.

	β-D-glucose		α-D-glucose		α-D-galactose		α-D-mannose	
	HB number	σ^2	HB number	σ^2	HB number	σ^2	HB number	σ^2
Total	9.81	1.42	9.06	1.57	8.99	1.43	9.05	1.59
Acceptor	5.43	1.17	4.92	1.28	4.88	1.18	4.95	1.27
Donor	4.38	0.72	4.20	0.79	4.22	0.79	4.12	0.82

Table S6. The average H-bonded distances (Å)

	β-D-glucose		α-D-glucose		α-D-galactose		α-D-mannose	
	Distance (Å)	σ^2	Distance (Å)	σ^2	Distance (Å)	σ^2	Distance (Å)	σ^2
O1H…O_w	1.784	0.163	1.811	0.160	1.784	0.158	1.791	0.159
O2H…O_w	1.823	0.161	1.831	0.169	1.812	0.170	1.850	0.180
O3H…O_w	1.869	0.178	1.871	0.192	1.850	0.175	1.833	0.168
O4H…O_w	1.796	0.154	1.834	0.169	1.885	0.185	1.827	0.164
O6H…O_w	1.811	0.154	1.829	0.166	1.829	0.164	1.864	0.191
O1…H_w	1.939	0.184	1.964	0.191	1.911	0.174	1.938	0.177
O5...H_w	1.993	0.190	2.003	0.199	1.920	0.170	1.990	0.195
O2...H_w	1.900	0.169	1.931	0.174	1.911	0.179	1.929	0.179
O3…H_w	1.866	0.174	1.907	0.182	1.886	0.173	1.898	0.164
O4…H_w	1.880	0.167	1.893	0.181	1.892	0.172	1.893	0.174
O6…H_w	1.881	0.151	1.919	0.159	1.894	0.165	1.872	0.168

Table S5 shows that β-D-glucose has the highest number of H-bonds. The H-bond for O1-H1...O_w is the shortest for all the studied monosaccharides. The average H-bond distance (Table S6) is significantly shorter when the sugar acts as an H-bond donor. The longest H-bond in all four cases is obtained for O5…H_wO_w. It is also found that the average H-bond distance for both donor and acceptor is the shortest for β-D-glucose.

7. <u>Dipole moment of water molecules</u>

Figure S10 shows the dependence of the dipole moment of water molecules that are not H-bonded to a sugar molecule or that are hydrogen-bonded as an acceptor or donor to the O1 atom of the β-D-glucose as a function of the number of H-bonds that can be assigned to this water molecule. The dipole moment of water molecules depends on H-bond numbers (almost linearly, at least in the 1-4 H-bonding range), and this dependence is similar to water molecules bound to sugars. Water molecules that are bound to the O1H group as an H-donor have a higher average dipole moment than water molecules bound to this group as a donor.

The average dipole moment of water molecules bound differently to the various oxygen species is given in Table 7. These data also support that the dipole moment of water molecules that bind to sugars significantly depends on how these molecules form H-bonds. It can be concluded that the dipole moment of water molecules is higher for all types of OH groups when this group forms a bond as an H-bond donor. Both α- and β-D-glucose have the highest dipole moment for O6 oxygen. One explanation could be that these water molecules can interact with the O5 ring oxygen.

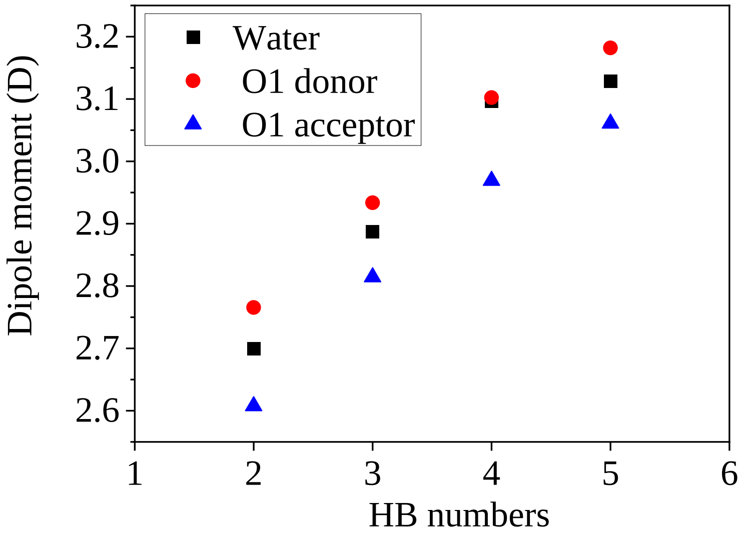

Figure S10. Average dipole moment (D) of water as a function of H-bonded neighbor numbers.

Table S7. Average dipole moment (D) of water connected to the OH and ring O.

	β-D-glucose		α-D-glucose	
	donor	acceptor	donor	acceptor
O1	3.05	2.91	3.01	2.93
O2, O3, O4	3.04	2.98	3.03	2.92
O5		2.91		2.95
O6	3.11	3.03	3.06	3.00
water	3.01		2.98	

8. Comparison between the experimental and theoretical hydration numbers of monosaccharides

Theoretically, monosaccharides composed of a hexapyranose ring can form 17, namely 5x3 (2 acceptors, 1 donor) +2 (2 acceptors) H-bonds with water. The hydration values obtained by various experimental techniques[24-28], which are mainly related to the non-dynamic properties of molecules in the hydration sphere vary between 3.5 and 8.4. NMR spectroscopy data show 10-11 water molecules around the monosaccharides[29]. On the other hand, the dielectric spectroscopic and mid-infrared data[30-31] suggest the presence of 21.0, 24.6 water molecules whose dynamic properties are significantly altered. Experiments based on light scattering, together with MD simulations[32], estimate the number of water molecules in the hydration sphere to be 14-15, which may well correspond to the number of water molecules located at a distance of 3.6 Å from the oxygen of the sugar molecule. Neutron diffraction measurements and their analysis by simulation methods assume about 15 water molecules[33-34]. Classical and ab initio

MD simulations have determined that the total hydrogen bond of water molecules is between 9 and 11[34-37]. Here, we would like to remark that this number significantly depends on the applied H-bond definition. In our AIMD simulation, the number of hydrogen-bonded water molecules is in the range of 10.7-11.4 for different types of monosaccharide if we apply the r(O···H) < 2.5 Å and the H-O…O angle < 30° definition, but it decreases to 9-9.8 with a stricter angular definition (20°). Using a simple distance definition, we can obtain about 13-14 water molecules with a reasonable agreement with the earlier results[32] supported by the light scattering experiment. Additionally, if the author takes into account the contact water molecules (hydrophobically bonded), the total hydration number is about 19-20.[36] This value is also in reasonable agreement with our results.

References

(1) Bakó, I.; Daru, J.; Pothoczki, Sz.; Pusztai, L.; Hermansson K. Effects of H-bond asymmetry on the electronic properties of liquid water – An AIMD analysis. J. Mol. Liq. 2019, 293, 111579. https://doi.org/10.1016/j.molliq.2019.111579

(2) Hardy, E. H.; Zygar, A.; Zeidler, M. D.; Holz, M.; Sacher, F. D. Isotope effect on the translational and rotational motion in liquid water and ammonia. J. Chem. Phys. 2001, 114, 3174. https://doi.org/10.1063/1.1340584

(3) Shi, R.; Russo, J.; Tanaka, H. Common microscopic structural origin for water's thermodynamic and dynamic anomalies. J. Chem. Phys. 2018, 149, 224502. https://doi.org/10.1063/1.5055908

(4) van der Spoel, D.; van Maaren, P. J.; Berendsen, H. J. C. A systematic study of water models for molecular simulation: Derivation of water models optimized for use with a reaction field. J. Chem. Phys. 1998, 108, 10220. https://doi.org/10.1063/1.476482

(5) Chandra, A.; Chowdhuri, S. Effects of hydrogen-bond environment on single particle and pair dynamics in liquid water. Proc. Indian Acad. Sci. (Chem. Sci.), 2001, 113, 5-6, 591–601.

(6) Paesani, F.; Voth, G. A. The properties of water: insights from quantum simulations. J. Phys. Chem. B 2009, 113, 5702. https://doi.org/10.1021/jp810590c

(7) Rezus, Y. L. A.; Bakker, H. J. On the orientational relaxation of HDO in liquid water. J. Chem. Phys. 2005, 123, 114502. https://doi.org/10.1063/1.2009729

(8) Winkler, R.; Lindner, J.; Bürsing, H.; Vöhringer, P. Ultrafast Raman-induced Kerr-effect of water: Single molecule versus collective motions. J. Chem. Phys. 2000, 113, 4674. https://doi.org/10.1063/1.1288690

(9) Hardy, E. H.; Zygar, A.; Zeidler, M. D.; Holz, M.; Sacher, F. D. Isotope effect on the translational and rotational motion in liquid water and ammonia. J. Chem. Phys. 2001, 114, 3174. https://doi.org/10.1063/1.1340584

(10) Ropp, J.; Lawrence, C.; Farrar, T.; Skinner, J. Rotational motion in liquid water is anisotropic: a nuclear magnetic resonance and molecular dynamics simulation study. J. Am. Chem. Soc. 2001, 123, 8047. https://doi.org/10.1021/ja010312h

(11) Ozkanlar, A.; Zhou, T.; Clark, A. E. Towards a unified description of the hydrogen bond network of liquid water: A dynamics based approach. J. Chem. Phys. 2014 141, 214107. https://doi.org/10.1063/1.4902538

(12) Martiniano, H. F. M. C.; Galamba, N. Insights on Hydrogen-Bond Lifetimes in Liquid and Supercooled Water. J. Phys. Chem. B 2013, 117, 16188−16195. https://doi.org/10.1021/jp407768u

(13) Guardia, E.; Skarmoutsos, I.; Masia, M. Hydrogen Bonding and Related Properties in Liquid Water: A Car–Parrinello Molecular Dynamics Simulation Study. J. Phys. Chem. B 2015, 119, 8926–8938. https://doi.org/10.1021/jp507196q

(14) Marti, J.; Padro, J. A.; Guardia, E. Molecular Dynamics Simulation of Liquid Water Along the Coexistence Curve: Hydrogen Bonds and Vibrational Spectra. J. Chem. Phys. 1996, 105, 639-649. https://doi.org/10.1063/1.471932

(15) Skarmoutsos, I.; Guardia, E. Effect of the Local Hydrogen Bonding Network on the Reorientational and Translational Dynamics in Supercritical Water. J. Chem. Phys. 2010, 132, 074502. https://doi.org/10.1063/1.3305326

(16) Lee, H. S.; Tuckerman, M. E. Structure of Liquid Water at Ambient Temperature from Ab Initio Molecular Dynamics Performed in the Complete Basis Set Limit. J. Chem. Phys. 2006, 125, 154507. https://doi.org/10.1063/1.2354158

(17) Dinga, Y.; Hassanali, A. A.; Parrinello, M. Anomalous water diffusion in salt solutions. PNAS 2014, 111, 3310-3315. https://doi.org/10.1073/pnas.1400675111

(18) Liu, J.; He, X., Zhang, J. Z. H.; Qi, L-W. Hydrogen-bond structure dynamics in bulk water: insights from ab initio simulations with coupled cluster theory. Chem. Sci. 2018, 9, 2065. https://doi.org/10.1039/C7SC04205A

(19) Busch, J.; Neumann, J.; Paschek, D. An exact a posteriori correction for hydrogen bond population correlation functions and other reversible geminate recombinations obtained from simulations with periodic boundary conditions. Liquid water as a test case. J. Chem. Phys. 2021, 154, 214501. https://doi.org/10.1063/5.0053445

(20) Gabrieli, A.; Sant, M.; Izadi, S.; Shabane, P. S.; Onufriev, A. V.; Suffritti, G. B. High-temperature dynamic behavior in bulk liquid water: A molecular dynamics simulation study using the OPC and TIP4P-Ew potentials. Front. Phys. 2018, 13(1), 138203. https://doi.org/10.1007/s11467-017-0693-7

(21) Chythra, J. N.; Mallajosyula, S. S. Impact of Polarization on the Ring Puckering Dynamics of Hexose Monosaccharides. J. Chem. Inf. Model. 2023, 63, 208-223. https://doi.org/10.1021/acs.jcim.2c01286.

(22) Hansen, H. S.; Hünenberger, P. H. A reoptimized GROMOS force field for hexopyranose-based carbohydrates accounting for the relative free energies of ring conformers, anomers, epimers, hydroxymethyl rotamers, and glycosidic linkage conformers. J Comput. Chem. 2011, 32, 998–1032. https://doi.org/10.1002/jcc.21675.

(23) Tvaroška, I.; Taravel, F. R.; Utille, J. P.; Carver, J. P. Quantum Mechanical and NMR Spectroscopy Studies on the Conformations of the Hydroxymethyl and Methoxymethyl Groups in Aldohexosides. Carbohydr. Res. 2002, 337, 53−367. https://doi.org/10.1016/S0008-6215(01)00315-9.

(24) Gharsallaoui, A.; Roge, B.; Genotelle, J.; Mathlouthi, M. Relationships between hydration number, water activity and density of aqueous sugar solutions. Food Chem. 2008, 106, 1443-1453. density https://doi.org/10.1016/j.foodchem.2007.02.047

(25) Shiio, H. Ultrasonic Interferometer Measurements of the Amount of Bound Water. Saccharides. J. Am. Chem. Soc. 1958, 80, 70-73. https://doi.org/10.1021/ja01534a020

(26) Galema, S. A.; Hoeiland, H. Stereochemical aspects of hydration of carbohydrates in aqueous solutions. 3. Density and ultrasound measurements. J. Phys. Chem. B 1991, 95, 5321-5326. https://doi.org/10.1021/j100166a073

(27) Bociek, S., Franks, F. Proton exchange in aqueous solutions of glucose. Hydration of carbohydrates. J. Chem. Soc. Faraday Trans. 1979, 1, 75, 262-270. https://doi.org/10.1039/F19797500262

(28) Furuki, T. Effect of molecular structure on thermodynamic properties of carbohydrates. A calorimetric study of aqueous di- and oligosaccharides at subzero temperatures. Carbohydr. Res. 2002, 337, 441. https://doi.org/10.1016/S0008-6215(01)00332-9

(29) Harvey, J. M.; Symons, M. C. R. Hydration of monosaccharides – Nmr-study. J. Solution Chem. 1978, 7, 571–586. https://doi.org/10.1007/BF00646035

(30) Shiraga, K.; Suzuki, T.; Kondo, N.; Tajima, T.; Nakamura, M.; Togo, H.; Hirata, A.; Ajito, K.; Ogawa, Y. Broadband dielectric spectroscopy of glucose aqueous solution: Analysis of the hydration state and the hydrogen bond network. J. Chem. Phys. 2015, 142, 234504. https://doi.org/10.1063/1.4922482

(31) Groot, C. C. M.; Bakker, H. J. A femtosecond mid-infrared study of the dynamics of water in aqueous sugar solutions. *Phys. Chem. Chem. Phys.* 2015, 17, 8449-8458. https://doi.org/10.1039/C4CP05431H

(32) Lupi, L.; Comez, L.; Paolantoni, M.; Perticaroli, S.; Sassi, P.; Morresi, A.; Ladanyi, B. M.; Fioretto, D. Hydration and Aggregation in Mono- and Disaccharide Aqueous Solutions by Gigahertz-to-Terahertz Light Scattering and Molecular Dynamics Simulations. J. Phys. Chem. B 2012, 116, 51, 14760–14767. https://doi.org/10.1021/jp3079869

(33) Mason, P. E.; Neilson, G. W.; Enderby, J. E.; Saboungi, M.-L.; Brady, J. W. Structure of Aqueous Glucose Solutions as Determined by Neutron Diffraction with Isotopic Substitution Experiments and Molecular Dynamics Calculations. J. Phys. Chem. B 2005, 109, 13104-13111. https://doi.org/10.1021/jp040622x.

(34). Mason, P. E.; Neilson, G. W.; Enderby, J. E.; Saboungi, M.-L.; Cuello, G.; Brady, J. W. Neutron diffraction and simulation studies of the exocyclic hydroxymethyl conformation of glucose. J. Chem. Phys. 2006, 125, 224505. https://doi.org/10.1021/ja051376l

(35) Lee, S. L.; Debenedetti, P. G.; Errington J. R. A computational study of hydration, solution structure, and dynamics in dilute carbohydrate solutions. J. Chem. Phys. 2005, 122, 204511. https://doi.org/10.1063/1.1917745

(36) Molteni, C.; Parrinello, M. Glucose in aqueous solution by first principles molecular dynamics. J. Am. Chem. Soc. 1998, 120, 2168−2171. https://doi.org/10.1021/ja973008q

(37) Suzuki, T. The Local Configurations in Sugar−Water Hydrogen Bonds. Phys. Chem. Chem. Phys. 2008, 10, 96−105. https://doi.org/10.1039/B708719E
